# Process evaluation of a school based physical activity related injury prevention programme using the RE-AIM framework

**DOI:** 10.1186/1471-2431-10-86

**Published:** 2010-11-23

**Authors:** Dorine CM Collard, Mai JM Chinapaw, Evert ALM Verhagen, Willem van Mechelen

**Affiliations:** 1 EMGO Institute for Health and Care Research and Department of Public & Occupational Health, van der Boechorststraat 7, 1081BT Amsterdam, The Netherlands

## Abstract

**Background:**

In general, only information regarding the effectiveness of an intervention programme is ever published. However, in recent years evaluating the translatability and feasibility of an intervention programme has become more important. Therefore, this paper presents the results of the evaluation of the iPlay programme aimed at preventing physical activity related injuries in primary school children.

**Methods:**

The iPlay programme targeted injuries gained through physical activity, and consisted of a teacher's manual, informative newsletters and posters, a website, and set exercises to be carried out during physical education (PE) classes. In order to evaluate the iPlay programme for translatability and feasibility, teachers, children and parents who participated in the iPlay programme filled out a questionnaire

The objective of this study is to describe the results of the process-evaluation of the iPlay programme based on the five dimensions of the RE-AIM framework.

**Results:**

The results showed that the participation rate of the children was 100% (reach). Nine percent of the schools who were invited to take part were willing to participate in the study (adoption rate). Teachers stated that they implemented the different elements of the programme partly as intended (implementation). The percentage of children and parents who followed the programme was less than expected. In addition, 52% of the teachers indicated that the current iPlay programme could become standard practice in their teaching routine (maintenance).

**Conclusion:**

The iPlay programme is a first start in the prevention of physical activity related injuries in children, but further improvements need to be made to the programme on the basis of this process evaluation.

**Trial registration:**

ISRCTN78846684; http://www.controlled-trials.com

## Background

Physical activity related injuries in primary school children are an unintentional consequence of their participation in physical activities. Nowadays, as children are encouraged to participate in physical activity in order to prevent obesity, for example, it is important to take the risk of injury into account. Therefore, injury prevention for children is important.

Due to the fact that school-based physical activity related injury prevention programmes are scarce, we developed and evaluated such a programme for children. This school-based program, called iPlay, was developed using the intervention mapping protocol [1] and evaluated through a cluster randomised controlled trial including over 2,200 children [2].

In general, only the controlled effects of an intervention programme are published. However, in recent years, the importance of evaluating the context in which interventions are implemented has been identified as critical. Evaluating factors such as translatability and feasibility are important, because if intervention programmes are not adopted to an adequate extent and then sustained, it is unlikely that these programmes will have any impact on public health. Nevertheless, these factors have received relatively little attention in the evaluation of interventions.

In order to evaluate the translatability and feasibility of the iPlay programme, the RE-AIM evaluation framework designed by Glasgow et al. [3] was used. This framework includes the following five dimensions: reach into the target population (i.e. children), the effectiveness of the intervention, extent of adoption in the target setting (i.e. schools), implementation, and maintenance of the intervention effects [4,5].

By evaluating these five dimensions, not only the translatability and feasibility of a programme can be identified, but also its limitations. In future research, these limitations can be improved upon.

This paper describes the process evaluation of a school based physical activity related injury prevention programme for Dutch primary school children based on the five dimensions of the RE-AIM framework.

## Methods

### Design, setting and participants

This evaluation was conducted as part of a cluster randomised controlled trial on the effectiveness of the iPlay programme for children aged 10-12 years old [2-6]. Dutch primary schools were randomly invited to participate in the study and assigned to an intervention or control group. The intervention group (20 schools) received the iPlay intervention programme aimed at injury prevention, whereas the control group (20 schools) followed the regular curriculum during the school year 2006-2007. Parents of the participating children received a passive informed consent form that explained the nature and procedure of the study. If parents and/or their children did not want to participate they could withdraw. The Medical Ethics Committee of the VU University Medical Centre approved the study design, protocols and informed consent procedures.

### iPlay programme

The iPlay programme was developed according to the intervention mapping protocol [1]. The intervention focused both on the children and their parents and consisted of newsletters, posters, exercises, a manual and a website.

Teachers were asked to distribute a monthly newsletter among their pupils containing information about injury prevention. The children were asked to read the newsletters attentively. Moreover, parents also received a newsletter each month with information about injury prevention and strategies intended to reduce the risk of injury to their child. They were also asked to read the newsletters attentively. Throughout the school year eight different newsletters were distributed among children and parents.

In addition to the newsletters, each month posters addressing the main topics of the intervention regarding injury prevention were displayed in the classroom in such a way that the children were able to see the posters at all times. There were also eight different posters that were displayed in the classroom throughout the school year. Children and their parents were encouraged to visit the website and to read more information about injury prevention.

In addition to the newsletters and posters, the iPlay programme included short recommended exercises aimed at improving the strength, speed, overall coordination and flexibility of the children. Teachers were asked to conduct the exercises during the first and last five minutes of each PE class, twice a week. There was a wide variety of exercises that the teachers could choose from. Furthermore, teachers received a manual with comprehensive information about the main goals of the iPlay programme, the time schedule for the intervention and explanations of the exercises.

### Data

The teachers, children and parents completed a questionnaire at the follow-up stage (June 2007), after the iPlay programme was completed. This questionnaire included questions designed to evaluate the potential of the intervention in terms of translation and feasibility.

The teachers, children and parents completed a questionnaire at follow-up (June 2007), after the iPlay programme was completed. This questionnaire included questions designed to evaluate the potential of the intervention in terms of translation and feasibility.

The questions focused on the implementation and maintenance of the iPlay programme. Teachers answered questions regarding their distribution of the newsletters and display of the posters; whether they drew attention to the newsletters and posters; how many times they performed the exercises and whether they performed the exercises as described; and whether the iPlay programme could become standard practice in their teaching routine' Children and parents were asked whether they had read the newsletters. We asked teachers, as well as children and parents about their opinion of the newsletters, posters and the overall iPlay programme.

In order to evaluate the iPlay programme on translatability and feasibility, we used five dimensions of the RE-AIM framework namely 'Reach', 'Effectiveness', 'Adoption', 'Implementation' and 'Maintanance'. The dimension 'effectiveness' is extensively described elsewhere in another manuscript [6]. Effectiveness was addressed at the participants' level and defined as the change in injury incidence density. This manuscript describes the results on the other four dimensions of the RE-AIM framework that are related to the translatability and feasibility of an intervention programme. The dimensions are outlined below.

Reach was defined as the absolute number and participation rate of children who attended schools that participated in the iPlay study. In order to assess the probability that the intervention could be generalised to the real world, the representativeness of the children was determined. Adoption was defined as the absolute number, proportion and representativeness of schools that were willing to participate in the iPlay study.

Implementation was addressed at the school level and at the participants' level. Implementation at school level was defined as the extent to which teachers successfully implemented the elements of the programme, including adherence to the implementation plan provided. Implementation at the participants' level was defined as the percentage of children and parents who followed the programme as intended. Furthermore, participants' satisfaction with the iPlay programme was assessed.

Maintenance was defined as the extent to which the iPlay programme became part of the standard teaching routine.

## Results

Complete questionnaires were returned by 96% of the teachers, 95% of the children and 59% of the parents.

### Reach

A total of 40 schools, comprising 2,210 children, were willing to participate in the study. Of these 2,210 children, only 2 children (0.1%) were unwilling to participate in the study. Baseline characteristics of the iPlay group compared to the total Dutch population of the same age are shown in Table 1. Children participating in this study did not differ from the general population of Dutch 10-12 year old children in terms of gender, body mass index (BMI) class and ethnicity. With regard to the parents of the children, 16 parents indicated that they were not willing to participate.

**Table 1:** Baseline characteristics of the iPlay group compared to the total Dutch population of the same age

CHARACTERISTICS		
	**iPlay-group N = 2.208**	**Total Dutch population N = 585.772**

Gender		
Boys (%)	50	51
Girls (%)	50	49

BMI class ^a^		
Normal weight (%)	82	86
Overweight and obese (%)	17	14
Unknown (%)	0.5	

Ethnicity		
Western (%)	79	85
Non-Western (%)	18	15
Unknown (%)	4	

^a^using the cut-off values described by Cole et al. [10]

BMI = body mass index

### Effectiveness

In order to evaluate the effectiveness of the iPlay programme, the programme was analysed in a randomized controlled trial conducted in the period 2006-2007. The results of the effectiveness of the iPlay programme on injury incidence density and injury severity are described in detail elsewhere [6]. In short, although not statistically significant, the results showed a consistently favourable intervention effect on injury incidence. Remarkably, the data showed that children who were less physically active benefitted to a greater extent from the iPlay programme.

### Adoption

All primary schools in the Netherlands were eligible for inclusion in the study. From the 7,000 primary schools throughout the Netherlands, 520 primary schools (7%) were randomly selected from a database and invited to participate in the iPlay study by means of an information flyer. The inclusion criteria for the primary schools were: 1) they had to be a regular primary school; 2) they had to provide PE lessons twice a week, and 3) they had to be willing to appoint a contact person for the duration of the study.

Of the 520 schools, 370 schools (71%) did not respond to the invitation, 105 schools (20%) were unwilling to participate and 45 schools (9%) were willing to participate in the study. The main reason why schools were not willing to participate was a lack of time (55%). Other reasons included 'already participating in another project' (8%), 'injury prevention is not relevant' (10%) or 'no interest' (8%). Schools that were unwilling to participate were not different from participating schools in terms of geographic location (urban versus rural areas) or the professional status of the PE teacher (certified/uncertified). A comparison of the participating and non-participating schools in terms of other variables (e.g. school resources or staff-to-children ratio) was not possible because information on those variables was lacking.

### Implementation

Almost all of teachers (96%) indicated that they had distributed all eight newsletters. Two thirds of the teachers drew attention to the newsletters in the classroom for an average of 11 ± 5.5 minutes per newsletter. Three quarters of the teachers displayed all eight posters. Sixty-eight percent drew attention to the posters (for an average of 7.5 ± 4.5 minutes per poster). All teachers had displayed the first poster in the first month. In the following months only one or two teachers had not displayed the respective poster (not the same teachers each month). Reasons for not displaying the posters were too busy or no space for the poster. Eight teachers had not displayed the last poster because they had not reach this stage.

Most of the teachers (71%) indicated that they taught the exercises most of the time. The exercises were performed during each lesson by 7% of the teachers. The main reasons for not performing the exercises were a lack of time and not enough space in the gymnasium. Teachers who performed the exercises did them for an average of 9.0 ± 2.3 minutes per PE class. Almost two-thirds of the teachers indicated that they had sometimes adapted the exercises. Furthermore, 69% of the teachers had read the manual completely. Half of the teachers had visited the website.

More than a quarter of the children (28%) had read all the newsletters they received, and 19% of the children did not read any of the newsletters. Approximately half of the children (53%) had read one or more of the newsletters. About the same percentages were reported regarding the posters. Sixteen percent of the children had visited the website.

Forty-one percent of the parents indicated that they had received all eight newsletters. Nine percent of the parents had not received any newsletters. Of the parents who received all of the newsletters, 55% indicated that they had read all of them. Five percent had not read any of the newsletters. Furthermore, nine percent of the parents had visited the website.

### Satisfaction with the iPlay materials

The majority of the teachers (76%) gave positive opinions of the iPlay newsletters and 15% were very positive. The teachers were also positive (68%) about the posters. The iPlay exercises were rated as very positive by 12% and as positive by 52%. All of the teachers indicated that the teacher's manual was clearly written. More than half of the teachers stated that the website included clear information about the elements of the iPlay programme. Twenty-seven percent thought that the website was a good method of support for the iPlay programme. More than half of the teachers (54%) indicated that it was easy to integrate the iPlay programme into their usual teaching routine. Two-thirds would recommend implementing the iPlay programme to other schools.

Sixty-five percent of the children indicated that they understood the iPlay newsletters. Fifty-three percent thought that the newsletters were educational and 35% thought that the newsletters were amusing. The score for the newsletters on a scale from 1 to 10 was 6.3 ± 2.4, where 1 is the lowest sore and 10 is the highest score. The overall score for the posters was 6.7 ± 2.3. Half of the children indicated that the exercises were fun to perform. Sixty-one percent thought that the exercises were easy to perform. Sixty percent of the children who visited the website indicated that the website was clear.

Eighty-five percent of the parents indicated that they had understood the newsletters. Fifty-three percent thought that the newsletters were educational and 72% thought the newsletters were fun to read. The overall score for the newsletters was 6.7 ± 1.2. Seventy-two percent of the parents who visited the website indicated that the website was clear.

### Maintenance

Approximately half of the teachers (52%) indicated that the iPlay programme would become standard practice in their teaching routine. Teachers indicated that extra time, commitment of the teachers, overall coordination of the programme and more variation in the iPlay programme were necessary for the successful implementation of the iPlay programme.

## Discussion

### Main findings

The results showed that the participation rate of the children was 100% (reach). The representativeness of the participating children was high regarding gender, BMI class and ethnicity compared to the source population (i.e. all Dutch children aged 10-12 years old). Nine percent of the schools who were invited to participate were willing to do so (adoption). Most of the schools (71%) did not respond to the invitation. Their main reason for not being willing to participate was a lack of time. It was not possible to describe the representativeness of the participating schools due to a lack of information. Teachers were positive about the iPlay programme and stated that they had implemented the elements of the programme as intended (implementation). The percentage of children and parents who followed the programme was less than expected. Children and parents were less positive about the elements of the iPlay programme than the teachers. Of the teachers, 52% indicated that the current iPlay programme could become standard practice in their teaching routine and 30% indicated that it could not become standard practice (maintenance).

A limitation of this study is that the questionnaires used for the evaluation were not validated and that the results are based on self-reports by the teachers, children and parents. Self-reports can lead to social desirability bias and over-reporting of compliance with the elements of the programme, and is a less valid means of assessing levels of implementation than other more objective methods such as observation during lessons [7,8]. However, observation is not feasible in large studies.

At the participants' level, maintenance is defined as the long-term effects of a programme. The long-term effects were not measured in the iPlay study.

The strength of this study is that the iPlay programme was not only evaluated on effectiveness on injury incidence, but also on translatability and feasibility. This is important because if interventions are not for example adequately adopted and sustained it is unlikely that the intervention will have a public health impact. Furthermore, the effectiveness of the iPlay programme can partly be explained by how the intervention was implemented.

We found a small intervention effect of iPlay on injury incidence. This process evaluation showed for example that not all children and their parents had read the newsletters In addition a lot of teachers adapted the exercises that were given twice a week during physical education lessons. Thanks to this process evaluation it is clear that the intervention was not fully implemented as intended and this may partly explain the small intervention effects we found.

### Strengths of the iPlay programme

The iPlay programme is, to our knowledge, the first school based physical activity related injury prevention programme for primary school children. The iPlay intervention showed small but promising effects in terms of the reduction of physical activity related injuries, especially in physically less active children. The results showed that injury prevention lessons should not only focus on children who participate in organised sports club activities, but on all children. Schools are an important setting because the reach into the student body is high. However, adoption and implementation of the programme need to be high.

Another strength of the iPlay programme is that it was developed in collaboration with teachers. In order to gain insight into the needs of the teachers and children and to design a feasible intervention programme, teachers were involved in the development process. As a result of this collaboration, the self-reported compliance and judgment of the teachers in the iPlay programme was high.

Furthermore, low-intensity interventions such as the iPlay programme are perhaps less effective than high-intensity interventions, but can also be delivered by less motivated and busy teachers and may therefore still make a health impact [9]. The iPlay programme is a low-intensity and 'easy to use' intervention program. Teachers indicated that it was easy to integrate the iPlay programme into their usual teaching routine.

### Limitations of the iPlay programme

Less than one out of the 10 schools who were invited was willing to participate in the iPlay study. It is likely that only highly motivated teachers participated in the study. Therefore, the effects of the iPlay programme cannot be generalized to all primary schools in the Netherlands. The most important reason for schools not being willing to participate was a lack of time. It must be mentioned that participating in a study requires much more time than participating in only an intervention because of the measurements during the school year. It is possible that this extra time taken by the evaluation process discouraged teachers to participate.

The iPlay programme was developed using the intervention mapping protocol [1]. This protocol provides a valuable checklist for the development of an intervention programme. Collaboration between the developers, the users of the intervention and the target population should lead to an 'ideal' intervention which is easy to implement. However, despite this collaboration, 30% of the teachers indicated that the current iPlay programme could not become a standard practice in their teaching routine. Apparently, the iPlay programme does not completely fit the needs of the teachers.

Another limitation of the iPlay programme is that less than half of the parents indicated that they received all eight newsletters, as mentioned before. The parents' newsletters were handed to the children. The children were asked to deliver the newsletter to their parents. The results showed that only a few parents received all of the newsletters. When improving the iPlay programme, one must think about another way to reach the parents.

Furthermore, children and parents were less positive about the iPlay programme than the teachers. This may be due to the fact that children and parents were less involved in the development of the iPlay programme. To improve the iPlay programme, and thereby the compliance of children and parents with the programme, the opinions of children and their parents about the iPlay materials should be taken into account.

## Conclusions

In this study, the RE-AIM evaluation framework was used in order to evaluate the iPlay programme on translatability and feasibility. Teachers indicated that the iPlay programme could be adapted into their teaching routine, but improvements to the iPlay programme are necessary. The compliance of children and parents with the programme should be improved by, for instance, including them in focus group interviews. The iPlay programme is a successful starting point in the prevention of physical activity related injuries in children, but the programme needs to be further improved on the basis of this process evaluation.

## List of Abbreviations

iPlay: Injury Prevention Lessons Affecting Youth; REAIM: Reach, Effectiveness, Adoption, Implementation, Maintenance; PE: Physical Education; BMI: Body Mass Index

## Competing interests

The authors declare that they have no competing interests.

## Authors' contributions

DC, MC, EV and WvM participated in the design of the study and contributed intellectual input into the main ideas of this paper. DC coordinated the implementation of the intervention, supervised the data-collection, performed the statistical analysis, and drafted the manuscript. DC had full access to all the data in the study and takes responsibility for the integrity of the data and the accuracy of the data analysis. All authors read and approved the final manuscript and declare that they have nothing to declare.

## Pre-publication history

The pre-publication history for this paper can be accessed here:

http://www.biomedcentral.com/1471-2431/10/86/prepub
